# Biochemical, Physiological and Transcriptomic Comparison between Burley and Flue-Cured Tobacco Seedlings in Relation to Carbohydrates and Nitrate Content

**DOI:** 10.3390/molecules22122126

**Published:** 2017-12-02

**Authors:** Yafei Li, Huijuan Yang, Dong Chang, Shuzhen Lin, Yuqing Feng, Jingjing Li, Hongzhi Shi

**Affiliations:** National Tobacco Cultivation & Physiology & Biochemistry Research Center, Henan Agricultural University, Zhengzhou 450002, China; liyafei@henau.edu.cn (Y.L.); huijuan@zju.edu.cn (H.Y.); cd411@outlook.com (D.C.); 18838933359@163.com (S.L.); 18838916870@163.com (Y.F.); qiyexueyl@126.com (J.L.)

**Keywords:** burley tobacco, chlorophyll, carbohydrate, nitrogen assimilation, nitrate

## Abstract

Burley tobacco is a genotype of chloroplast-deficient mutant with accumulates high levels of tobacco-specific nitrosamines (TSNAs) which would induce malignant tumors in animals. Nitrate is a principle precursor of tobacco-specific nitrosamines. Nitrate content in burley tobacco was significantly higher than that in flue-cured tobacco. The present study investigated differences between the two tobacco types to explore the mechanisms of nitrate accumulation in burley tobacco. transcripts (3079) related to the nitrogen and carbon metabolism were observed. Expression of genes involved in carbon fixation, glucose and starch biosynthesis, nitrate translocation and assimilation were significantly low in burley tobacco than flue-cured tobacco. Being relative to flue-cured tobacco, burley tobacco was significantly lower at total nitrogen and carbohydrate content, nitrate reductase and glutamine synthetase activities, chlorophyll content and photosynthetic rate (Pn), but higher nitrate content. Burley tobacco required six-fold more nitrogen fertilizers than flue-cured tobacco, but both tobaccos had a similar leaf biomass. Reduced chlorophyll content and photosynthetic rate (Pn) might result in low carbohydrate formation, and low capacity of nitrogen assimilation and translocation might lead to nitrate accumulation in burley tobacco.

## 1. Introduction

Burley, known as yellow green leaf color tobacco, is a chloroplast-deficient mutant tobacco type with reduced pigment content [1]. Little has been reported on the effect of chloroplast-deficient mutant in burley tobacco. Leaf chlorophyll status is used to evaluate plant photosynthesis and nutritional stress [2]. The amount of nitrogen fertilizers used on burley tobacco was almost 3–5 times more than that on flue-cured tobacco, but the yield between them was not significantly different [3], indicating a much lower nitrogen utilization efficiency by burley tobacco. The nitrate content in air-cured burley tobacco was at least 50 times greater than that in flue-cured tobacco [4] and was regarded as an important cause of high tobacco-specific nitrosamines (TSNAs) formation in burley tobacco than that in flue-cured tobacco. Although it is well known that burley tobacco has high levels of nitrate, little is known about the mechanism of high level accumulation of nitrate in cultivation.

Nitrate (NO_3_^−^) is one of the major nitrogen sources being taken up by plants, which can accumulate to a high concentration in plant cell vacuoles if it is not reduced, reutilized or transported into the cytoplasm [5]. Consumption of nitrate could be harmful to humans. On the one hand, nitrate could be reduced to nitrite which has a priority of being re-oxidized to nitrate by oxyhemoglobin in the bloodstream with the resultant formation of methemoglobin. The capacity of blood to deliver oxygen to the body tissues would be impaired [6]. This condition is referred to as methemoglobinemia and is harmful to growing children and adults. On the other hand, nitrate was one of the principle precursors contributing to the formation and accumulation of TSNAs which would induce malignant tumors in mice, rats and hamsters [7]. In recent years, reducing nitrate accumulation has been an important research strategy for reduction of TSNA formation in tobacco.

Many factors such as nitrogen application, tobacco types and varieties, cultivation environment and conditions were all related to nitrate accumulation [8]. An increased amount of nitrogen fertilizer application generally gave rise to higher levels of nitrate [9]. Low nitrogen efficiency varieties usually had higher nitrate accumulation than high nitrogen efficiency varieties under the same nitrogen levels [10]. Differences in nitrate accumulation among varieties are due to their differential capacities in absorbing, reducing and assimilating nitrate [11]. High assimilation was regarded as a main contributor to low nitrate concentration in the lamina [12]. Nitrate reductase (NR) and glutamine synthetase (GS) are important in nitrogen metabolism, and their activities have significant effects on nitrate accumulation in plants. NR is responsible for the reduction of nitrate to nitrite in the cytoplasm, which is the first step of nitrogen assimilation and utilization. GS can catalyze the first step in the conversion of inorganic nitrogen (ammonium) into organic form (glutamine); NR and GS play important roles in nitrogen reutilization, nitrogen assimilation and photorespiratory N cycle [13]. Activities of NR and GS are all related to the chloroplast. NR is very low in pigment-deficient leaves of chloroplast-ribosome-deficient mutants [14]. About 40% of GS in the leaf cells is in the chloroplasts [15], since chloroplasts supply energy by photosynthesis for nitrogen metabolism in plants [16].

In the present study, pot experiments were carried out to investigate the differences in carbohydrate and nitrate accumulation between burley tobacco and flue-cured tobacco on the basis of plant physiology, biochemistry and the plant transcriptome. RNA sequencing technology was used to analyze the differences in nitrogen and carbon metabolism between the two types to explore the reasons causing higher nitrate accumulation and lower carbohydrate content in burley tobacco. Enzymatic activities of NR and GS, pigment content, photosynthetic trait, nitrogen and carbohydrate content were also studied aiming to investigate the phenotype differences in photosynthetic rate, capacity of nitrogen assimilation, carbohydrates and nitrate accumulation between burley tobacco and flue-cured tobacco seedlings.

## 2. Results

**Figure 1:**
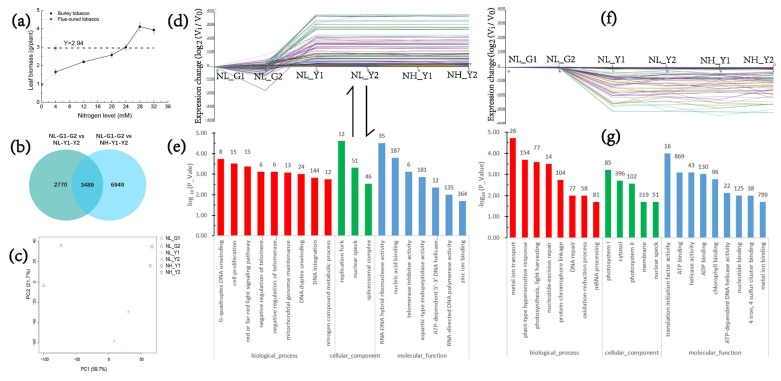
Transcriptome analysis strategies and GO enrichment of target DEGs. (**a**) Leaf biomass in burley tobacco and flue-cured tobacco; (**b**) Venn diagram of DEGs of flue-cured tobacco (NL_G1_G2) vs burley tobacco (NL_Y1_Y2) at the same nitrogen application level and flue-cured tobacco (NL_G1_G2) vs. burley tobacco (NH_Y1_Y2) at the same leaf biomass accumulation condition; (**c**) PCA analysis of treatments; (**d**) Genes represented in Profile 73; (**e**) GO enrichment in Profile 73; (**f**) Genes represented in Profile 38; (**g**) GO enrichment in Profile 38. NL_G1: low nitrogen level, HD; NL_G2: low nitrogen level, Z100; NL_Y1: low nitrogen level, TN90; NL_Y2: low nitrogen level, TN86; NH_Y1: high nitrogen level, TN90; NH_Y2: high nitrogen level, TN86.

Experiments of varying nitrogen application rates on burley tobacco were performed in the earlier preparation stage. The results showed that leaf biomass between the two tobacco types was equal at 24 mmol/L nitrogen level for burley tobacco and 4 mmol/L nitrogen level for flue-cured tobacco during seedling stage (Figure 1a). Differences between the two tobacco types at the same nitrogen application levels and at the same leaf biomass condition were then analyzed.

### 2.1. RNA-Seq Statistics, and Molecular Analysis on Nitrogen and Carbon Metabolism

Eighteen cDNA libraries (2 varieties * 3 treatments * 3 biological replicates) were prepared to analyze the differences in nitrogen and carbon metabolism between burley tobacco and flue-cured tobacco seedlings. After removing sequencing adaptors and low quality reads, we obtained 74.79 M reads in tobacco leaves. In all, an average mapping ratio of 85.15% was mapped to the reference genome (ftp://ftp.solgenomics.net/genomes/Nicotiana_tabacum/assembly/Ntab-K326_AWOJ-SS.fa.gz), and an average mapping ratio of 4.10% reads had multiple locations, and 81.08% of them had unique location in that genome.

In the process of DEGs screening, fold change (FC) > 2 or FC < 0.5, *p*-value < 0.05, was used as threshold to determine the significance of gene expression differences between flue-cured tobacco and burley tobacco; meanwhile, genes of DEGs, both of flue-cured tobacco and burley tobacco, with FPKM < 1 were removed. FC is radio of FPKM between burley tobacco (FPKM, average of TN90 and TN86) and flue-cured tobacco (FPKM, average of HD and Z100). To determine the stable transcriptional difference between flue-cured tobacco (HD and Z100) and burley tobacco (TN90 and TN86), GO and KEGG classifications were implemented for genes belonging to certain expression profiles screened by STEM. Genes in burley tobacco varieties were mostly down-regulated and up-regulated in profile 38 (genes expression pattern: NL_G1, NL_G2, NL_Y1, NL_Y2, NH_Y1, NH_Y2 = 0, 0, −1, −1, −1, −1) and profile 73 (genes expression pattern: NL_G1, NL_G2, NL_Y1, NL_Y2, NH_Y1, NH_Y2 = 0, 0, 1, 1, 1, 1), respectively, compared with the flue-cured tobacco varieties at the same nitrogen application and the same leaf biomass accumulation condition (Figure 1b–f). In profile 38, we observed high values for categories involved in gene ontology biological processes (GO-BP), such as metal ion transport (GO:0030001), plant-type hypersensitive response (GO:0009626), photosynthesis, light harvesting (GO:0009765), protein-chromophore linkage (GO:0018298), oxidation-reduction process (GO:0055114). Genes mostly involved in gene ontology cellular component (GO-CC), such as photosystem I (GO:0009522), cytosol (GO:0005829), photosystem II (GO:0009523). In profile 73, we observed high values for categories involved in gene ontology biological processes (GO-BP), such as G-quadruplex DNA unwinding (GO:0044806), cell proliferation (GO:0008283), red or far-red light signaling pathway (GO:0010017), negative regulation of telomere maintenance via telomerase (GO:0032211), negative regulation of telomerase activity (GO:0051974), DNA duplex unwinding (GO:0032508), DNA integration (GO:0015074). The genes mainly involved in gene ontology cellular component (GO-CC), such as replication fork (GO:0005657), nuclear speck (GO:0016607), spliceosomal complex (GO:0005681).

Transcripts (3079) were mainly represented in carbon and nitrogen metabolism. DEGs (207) correlated with response to nitrate (GO:0010167), nitrate transport (GO:0015706), nitrate assimilation (GO:0042128), starch and sucrose metabolism (Ko00500) were preferentially observed. Twenty three (down-regulated) and ten (up-regulated) target DEGs between the two types of tobacco were significantly represented in profile 38 and profile 73, respectively (Tables S1 and S2).

**Figure 2:**
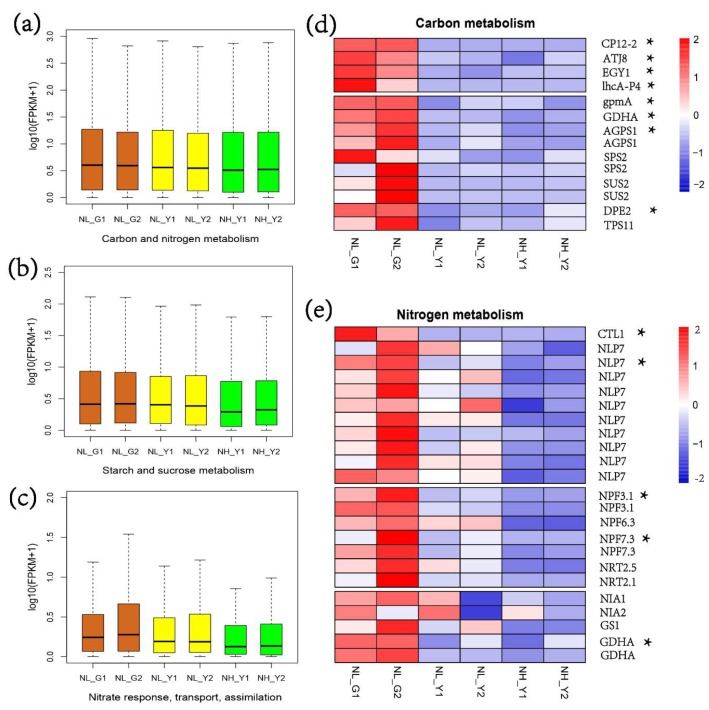
Expressed pattern analysis of genes involved in nitrogen and carbon metabolism in flue-cured tobacco and burley tobacco. (**a**) Primary transcripts correlated with carbon and nitrogen metabolism; (**b**) Starch and sucrose metabolism; (**c**) Nitrate response, transport and assimilation; (**d**) Expression pattern of genes related to carbon metabolism; (**e**) Expression pattern of genes related to nitrogen metabolism. NL_G1: low nitrogen level, HD; NL_G2: low nitrogen level, Z100; NL_Y1: low nitrogen level, TN90; NL_Y2: low nitrogen level, TN86; NH_Y1: high nitrogen level, TN90; NH_Y2: high nitrogen level, TN86. Box-whisker plot represents dispersity of minimum, first quartile, median, third quartile in genes expression level of treatments. Y-axis represents normalized expression level (log 10 (FPKM + 1)). Brown represents genes expression pattern of flue-cured tobacco grown at low nitrogen level. Yellow represents genes expression pattern of burley tobacco grown at low nitrogen level. Green represents genes expression pattern of burley tobacco grown at high nitrogen level. Date are means of three biological replications. Symbol * indicated that gene expressed pattern was significant enrichment in profile (0, 0, −1, −1, −1, −1) performed by STEM. The same gene labels represent the different transcripts of the same gene.

Figure 2a,b show that carbon and nitrogen metabolism, starch and sucrose metabolism (Ko00500) were significantly lower in burley tobacco than in flue-cured tobacco, which was consistent with the low carbohydrate concentration in burley tobacco. The expression levels of genes involved in responding to light (CP12-2, ATJ8 and EGY1), antenna proteins (lhcA-P4), starch and sucrose metabolism (GDHA, SUS2) were consistently suppressed at different nitrogen application levels in burley tobacco (Figure 2d). On average, gene expression levels involved in the responses to nitrate (GO:0010167), nitrate transport (GO:0015706) and nitrate assimilation (GO:0042128) were lower in burley tobacco than in flue-cured tobacco (Figure 2c). To explore the cause for higher nitrate accumulation in burley tobacco, we analyzed the differences in gene expression patterns of nitrogen metabolism between burley tobacco and flue-cured tobacco. The results showed that genes of CTL1, NLP7, NPF3.1 and NPF7.3 were consistently and significantly suppressed under both low and high nitrogen application treatments in burley tobacco (Figure 2e), which might be the cause for nitrate accumulation in burley tobacco.

**Figure 3:**
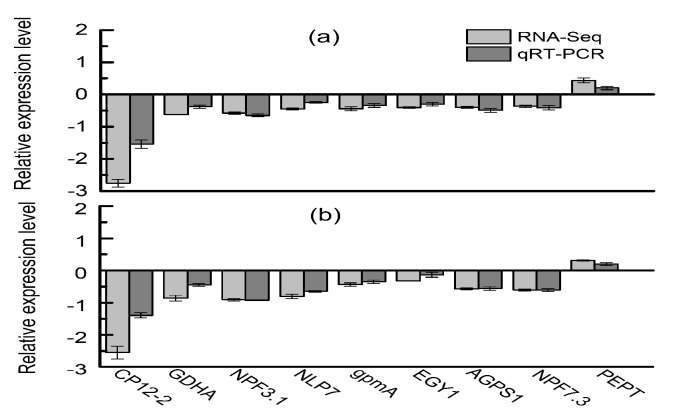
RNA-seq results confirmed by quantitative qRT-PCR. (**a**) qRT-PCR validation for nine selected genes in flue-cures tobacco and burley tobacco at same nitrogen level; (**b**) qRT-PCR validation for nine selected genes in flue-cures tobacco and burley tobacco at same leaf biomass condition. Error bars represent standard error (*n* = 6).

Eight down-regulated and one up-regulated unigenes were selected for qRT-PCR analysis to verify the reliability and accuracy of the transcriptome date. L25, a highly expressed and widely used reference genes, was used as an internal control. For most of these genes, their expression patterns were highly consistent both in qRT-PCR and transcriptome analyses (Figure 3a,b).

### 2.2. Differences in Pigment Content and Photosynthesis between the Two Types

**Figure 4:**
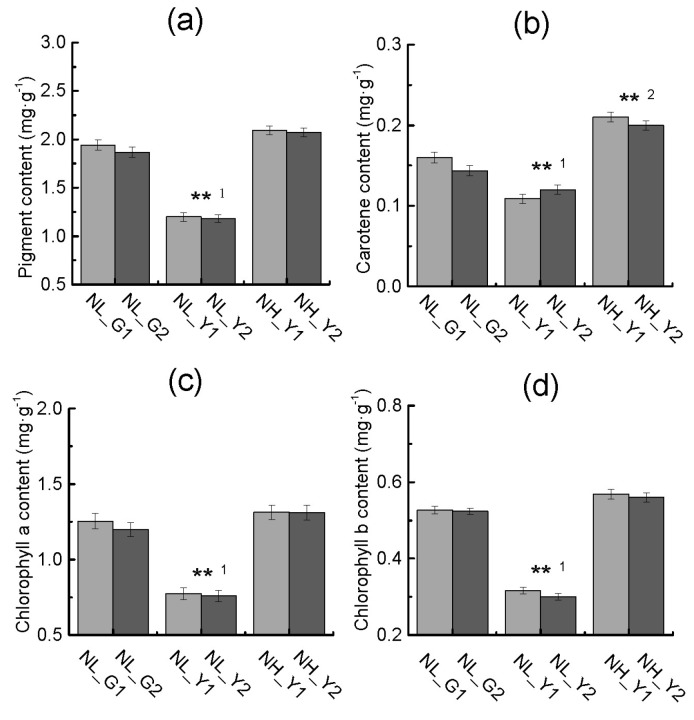
Differences in pigment content (**a**), carotene content (**b**), chlorophyll a (**c**), chlorophyll b (**d**) in flue-cured tobacco and burley tobacco at the same nitrogen level and same leaf biomass accumulation condition. NL_G1: low nitrogen level, HD; NL_G2: low nitrogen level, Z100; NL_Y1: low nitrogen level, TN90; NL_Y2: low nitrogen level, TN86; NH_Y1: high nitrogen level, TN90; NH_Y2: high nitrogen level, TN86. Error bars indicate standard error of the means (*n* = 3). Symbols ** ^1^ indicate that the significant differences between flue-cured tobacco and burley tobacco at the same nitrogen application are at 0.01, respectively. Symbols ** ^2^ indicate that the significant differences between flue-cured tobacco and burley tobacco at the same leaf biomass accumulation condition are at 0.01, respectively.

**Figure 5:**
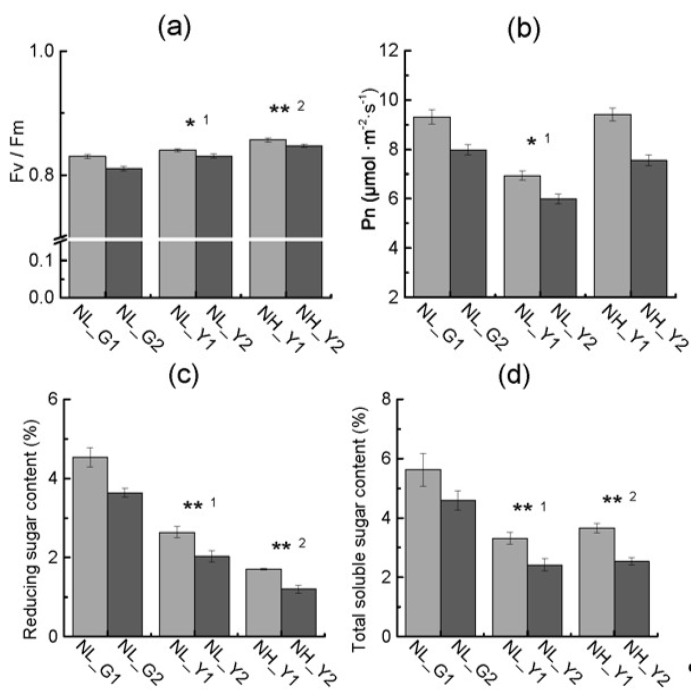
Differences in Fv/Fm (**a**), Pn (**b**), reducing sugar content (**c**), total soluble sugar content (**d**) in flue-cured tobacco and burley tobacco at the same nitrogen level and same leaf biomass accumulation condition. Fv/Fm: Maximum quantum yield of PS II photochemistry; Pn: Photosynthetic rate. NL_G1: low nitrogen level, HD; NL_G2: low nitrogen level, Z100; NL_Y1: low nitrogen level, TN90; NL_Y2: low nitrogen level, TN86; NH_Y1: high nitrogen level, TN90; NH_Y2: high nitrogen level, TN86. Error bars of chlorophyll a fluorescence and photosynthesis rate indicate standard error of the means (N = 20, “N” means the number of individuals), and error bars of reducing sugar content and total soluble sugar content indicate standard error of the means (*n* = 3, three biological replicates). Symbols ** ^1^ and * ^1^ indicate that the significant differences between flue-cured tobacco and burley tobacco at the same nitrogen application are at 0.01 and 0.05, respectively. Symbols ** ^2^ indicate that the significant differences between flue-cured tobacco and burley tobacco at the same leaf biomass accumulation condition are at 0.01, respectively.

Figure 4 shows that pigment, chlorophyll a, chlorophyll b and carotene contents in burley tobacco increased with the increase of nitrogen application. Pigment, chlorophyll a content, chlorophyll b and carotenoid contents were significantly lower in burley tobacco (mean of TN90 and TN86) than those in flue-cured tobacco (mean of HD and Z100) at the same nitrogen application level. Fv/Fm was significantly higher in burley tobacco than that in flue-cured tobacco at the same nitrogen level and at the same leaf biomass accumulation, respectively. Pn was always lower in burley tobacco than that in flue-cured tobacco (Figure 5a,b). Lower pigment content and weaker photosynthesis may have an influence on carbon fixation and lead to low carbohydrate accumulation in burley tobacco.

### 2.3. Differences in Enzymes Activities Correlated with Nitrogen Metabolism

**Figure 6:**
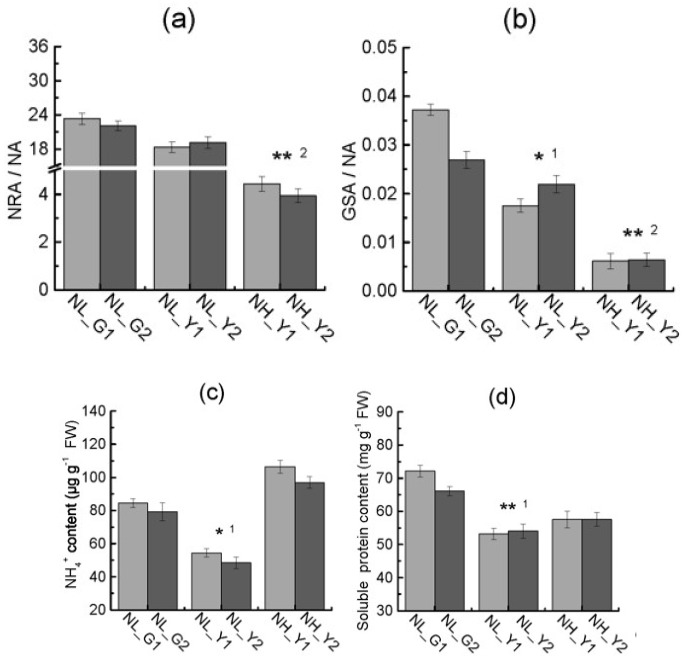
Differences in NRA/NA (**a**), GSA/NA (**b**), NH_4_^+^ content (**c**) and soluble protein content (**d**) between the two types at the same nitrogen level and same leaf biomass accumulation condition. NA: Nitrogen application level; NRA/NA: Ratio of nitrate reductase activity to nitrogen application level; GSA/NA: Ratio of glutamine synthetase activity to nitrogen application level. NL_G1: low nitrogen level, HD; NL_G2: low nitrogen level, Z100; NL_Y1: low nitrogen level, TN90; NL_Y2: low nitrogen level, TN86; NH_Y1: high nitrogen level, TN90; NH_Y2: high nitrogen level, TN86. Error bars indicate standard error of the means (*n* = 3). Symbols ** ^1^ and * ^1^ indicate that the significant differences between flue-cured tobacco and burley tobacco at the same nitrogen application are at 0.01 and 0.05, respectively. Symbols ** ^2^ indicate that the significant differences between flue-cured tobacco and burley tobacco at the same leaf biomass accumulation condition are at 0.01, respectively.

NR and GS both play important roles in the process of nitrogen metabolism. In order to understand whether the key enzymes involved in nitrogen metabolism were the key difference between burley tobacco and flue-cured tobacco on nitrogen and nitrate accumulation, we measured the activities of NR, GS and their reaction products in burley tobacco and flue-cured tobacco leaves (Figure 6a,b). The ratios of nitrate reductase activity to nitrogen application level (NRA/NA) and glutamine synthetase activity to nitrogen application level (GSA/NA) were lower in burley tobacco than in flue-cured tobacco at the same nitrogen level. Furthermore, the ratios of NRA/NA and GSA/NA in burley tobacco were significantly low at the same leaf biomass accumulation, compared with that in flue-cured tobacco. NH_4_^+^ content in burley tobacco was dramatically lower than that in flue-cured tobacco at the same nitrogen application level, but the content of NH_4_^+^ in burley tobacco was slightly higher than that in flue-cured tobacco at the same leaf biomass accumulation (Figure 6c). In contrast, soluble protein content in burley tobacco at low and high nitrogen application levels were always lower than those in flue-cured tobacco (Figure 6d). In particular, the weak ability of nitrogen reutilization on nitrogen reduction and nitrogen assimilation between the two types was in line with analysis of transcriptomic results.

### 2.4. Differences in Carbon and Nitrogen Compounds between Flue-Cured and Burley Tobacco

**Figure 7:**
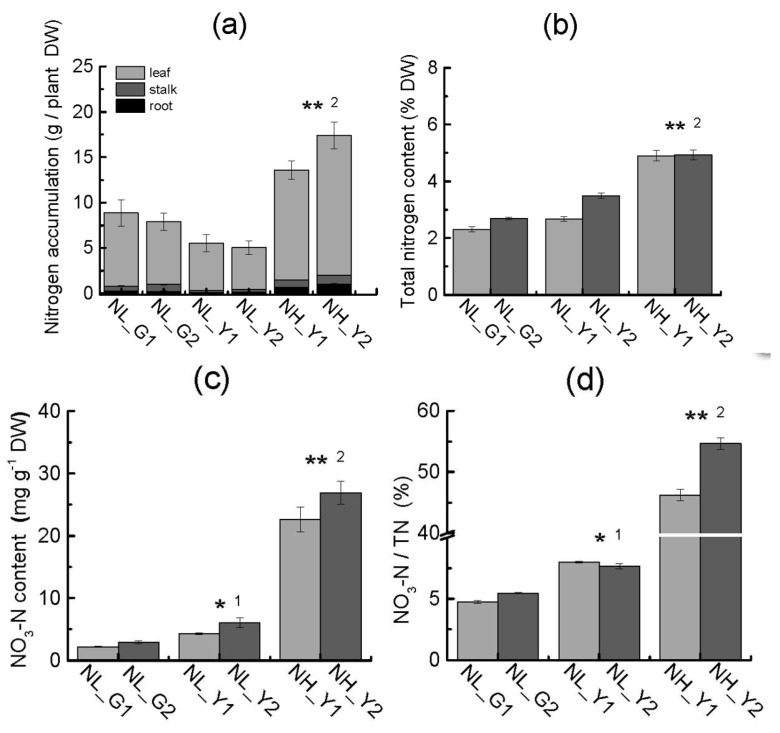
Differences in nitrogen compound between the two types at the same nitrogen level and same leaf biomass accumulation condition. (**a**) Nitrogen accumulation per plant; (**b**) Total nitrogen content (TN); (**c**) NO_3_-N content; (**d**) Ratio of NO_3_-N to total nitrogen content (TN) between the two types of different treatments. NL_G1: low nitrogen level, HD; NL_G2: low nitrogen level, Z100; NL_Y1: low nitrogen level, TN90; NL_Y2: low nitrogen level, TN86; NH_Y1: high nitrogen level, TN90; NH_Y2: high nitrogen level, TN86. Error bars indicate standard error of the means (*n* = 3). Symbols * ^1^ indicate that the significant differences between flue-cured tobacco and burley tobacco at the same nitrogen application are at 0.05, respectively. Symbols ** ^2^ indicate that the significant differences between flue-cured tobacco and burley tobacco at the same leaf biomass accumulation condition are at 0.01, respectively.

Nitrogen and carbohydrate contents were different between burley tobacco and flue-cured tobacco (Figure 5c,d and Figure 7a–d). The total soluble sugar and reducing sugar content in burley tobacco were lower than those in flue-cured tobacco under the same nitrogen level or same leaf biomass accumulation level. The reducing sugar content was lower at high nitrogen application rates than that at low nitrogen application levels for burley tobacco. The total nitrogen content and nitrogen accumulation in burley tobacco were significantly higher than those in flue-cured tobacco at the same nitrogen application or the same leaf biomass accumulation condition. Nitrogen accumulation per plant was lower in burley tobacco than that in flue-cured tobacco at the same nitrogen application, indicating that burley tobacco may have lower ability of nitrogen absorption than flue-cured tobacco. In addition, NO_3_-N content and the ratio of NO_3_-N/total nitrogen content (TN) in burley tobacco under high and low nitrogen application treatment were significantly higher than those in flue-cured tobacco. Differences in nitrogen metabolism between the two types might be due to the different ability of nitrogen reutilization in the leaf, leading to nitrate accumulation in burley tobacco.

### 2.5. Correlation Analysis

The correlation coefficients between carbon nitrite and nitrogen and carbon metabolism and metabolites are listed in Table 1 and Table 2. The results showed that NO_3_-N and ratio of NO_3_-N to total nitrogen content (TN) had significantly negative correlation with enzyme activities of nitrogen assimilation and photosynthetic rate, indicating that capability of nitrogen assimilation and photosynthesis efficiency might have huge effects to nitrate accumulation in tobacco. In contrast, total soluble sugar content had positive correlation with the enzyme activities of nitrogen assimilation and photosynthetic rate, indicating that carbohydrate formation in tobacco were mainly co-determined by carbon and nitrogen metabolism. A positive correlation was also observed between leaf biomass and enzyme activities of nitrogen assimilation and photosynthetic rate, while there was significant correlation with pigment content.

## 3. Discussion

The objectives of this research were to investigate the physiological, biochemical and transcriptome differences in nitrogen and carbon metabolism between flue-cured tobacco and burley tobacco, and to explore the reasons for low carbohydrate formation and high nitrate accumulation in burley tobacco. The results from this study demonstrated that genes related to light stimulus responses, carbon fixation in photosynthesis and starch and sucrose biosynthesis, response to nitrate, nitrate transport and nitrate assimilation were significantly suppressed in burley tobacco at both low and high nitrogen applications (Figure 2a–e).

Burley is one tobacco type characteristic of yellow–green leaf, whose pigment content is obviously lower than that of flue-cured tobacco (Figure 4a–d). Photosynthesis and its products in burley tobacco were all decreased (Figure 5b–d). Some key genes related to chlorophyll biosynthetic process and photosynthesis were significantly down-regulated in burley tobacco, including Protein CP12, which is believed to be an oxygenic photosynthetic organisms [17], EGY1 gene, which has pleiotropic effects both on chloroplast development and on ethylene-dependent gravitropism of light-grown hypocotyls [18], ATJ8 gene, which has effects on photosynthesis by diminishing photosynthetic efficiency and destroying PS II complex stability [19]. Gene of lhcA-P4 encode key subunit of light-harvesting complex which could convert light energy into chemical energy [20]. The down-regulation of these genes might decrease the chlorophyll formation and photosynthesis efficiency in burley tobacco. Sucrose synthase (SUS) plays a dominant role in generating precursors for starch biosynthesis [21], and it catalyzes and controls the flow of carbon into starch biosynthesis [22]. Sucrose-phosphate synthase (SPS) plays the role of rate-limiting steps in sucrose synthesis in higher plant [23]. ADPG pyrophosphorylase (AGPase) exclusively catalyzes the synthesis of ADPG and acts as the major limiting step of the gluconeogenic process, which could serve as universal glucosyl donor in the reaction process of starch synthase [24]. Genes SUS2, SPS2, AGPS1 were constantly down-regulated both in burley tobacco at the low and high nitrogen application, which was consistent with low carbohydrate formation in burley tobacco.

With six times more nitrogen applied, the NO_3_^−^ content in burley tobacco seedlings increased by 4.79-fold (Figure 7c). Nitrogen application in commercial burley production was almost 3–4 times higher than that in flue-cured tobacco, while NO_3_^−^ content in burley cured leaves was tens or hundreds of times greater than that in flue-cured tobacco [25]. In general, nitrogen accumulation in burley tobacco was lower than that in flue-cured tobacco at the same nitrogen application level (Figure 7a), suggesting that nitrate accumulation in burley tobacco leaf were not due to the efficient root nitrogen uptake but due to the lower ability of nitrogen utilization in leaf. CTL1 was essential for response to nitrate, regulating root architecture, normal plant growth and development [26]. Genes involved in response to nitrate (NLP7, CTL1) in leaf were significantly suppressed, suggesting that more nitrate needed to transport into the leaf to fulfill nitrogen metabolism in burley tobacco. Hence, much more nitrogen fertilizer would be required to meet the requirement of normal plant development of burley tobacco.

Both genes of high-affinity nitrate transporters (NRT2.1 and NRT2.5) and genes of low-affinity nitrate transporters (NPF3.1 and NPF7.3) were significantly down-regulated in burley tobacco leaf, which would in turn decrease nitrate transport ability, making nitrate translocation more difficult, and would lead to nitrate accumulation once it was stored in leaves [27]. NPF3.1 and NPF7.3 are low-affinity proton-dependent bidirectional nitrate transporter that are involved in nitrate loading into xylem but not in nitrate uptake [28]. NRT2.1 and NRT2.5 belong to high-affinity nitrate transporter, and are involved in nitrate transport too [29]. Both enzymatic activities (NRA, GSA) and expression levels of genes (NIA1, NIA2, GS1, GDHA) involved in nitrogen assimilation were lower in burley tobacco than those in flue-cured tobacco. Activities of NR and GS had great influence on nitrate concentration in higher plants, and the NR was considered as a limiting factor for nitrogen assimilation [30]. Activity of GS and soluble protein content were significantly lower in burley tobacco than those in flue-cured tobacco at the same nitrogen application level, indicating that the lower capacity of nitrogen assimilation in burley tobacco might lead to its higher nitrate accumulation in view of plant physiology, biochemistry and transcriptome.

NLP7, one major transcription factor, is a positive regulator of nitrate-induced expression of N-related genes through post-translation regulation, such as the genes NRT2.1, NITR2:1, NIA1, NIR1 [31]. NLP7 was reported to be involved in response to nitrate, nitrate assimilation, stomatal movement and DNA binding [32], and this gene expression level was consistently and significantly hampered in burley tobacco at low and high nitrogen levels (Figure 2e). Activities of NR and GS and total protein content in *Arabidopsis* NLP7 mutants were significantly reduced but nitrate content was significantly increased, the similar results were observed in burley tobacco [32]. In addition, chlorophyll content, photosynthesis and carbohydrates increased in NLP7-overexpressing and mutant plants [32]. The suppressed transcription factor NLP7 might be one key reasons for low carbohydrate formation and high nitrate accumulation in burley tobacco. The interesting candidates need to be further investigated in the future. 

In Conclusion, differences in carbohydrate and nitrate accumulation between burley tobacco and flue-cured tobacco were significant. Carbohydrate concentration in burley tobacco was significantly lower than that in flue-cured tobacco, which was closely associated with low pigment content and photosynthetic efficiency in burley tobacco. Nitrate accumulation was significantly greater in burley tobacco than in flue-cured tobacco. Aspects such as weak response to nitrate, weak nitrogen assimilation and nitrate translocation may combine to generate the phenotype of high nitrate accumulation in burley tobacco. The above analysis on the differences in nitrogen and carbon metabolism between burley tobacco and flue-cured tobacco in view of plant physiology, biochemistry and transcriptome clarify the direction for decreasing nitrate accumulation and improving nitrogen efficiency of burley tobacco in the future.

## 4 Material and Methods

### 4.1. Plant Material and Growth Conditions

Experiments were conducted on substrate culture in greenhouse that maintained a temperature of 23 ± 2 °C. Two types of burley tobacco and flue-cured tobacco were found to be significantly different in nitrate content in our former experiments [33]. Seeds were sterilized with 2% (*v*/*v*) sodium hypochlorite for 5 min twice. Seeds were sown in a floating system. Until having four to five permanent leaves (sown 40 days later), seedlings were washed with distilled water. Seedlings were transplanted in 25 cm × 30 cm (diameter × depth) plastic pots (plant/pot). In preliminary tests, seedlings being transplanted in plastic pots were treated with different nitrogen rates (0, 4, 12, 20, 24, 28, 32 and 36 mmol/L, treated 10 days) in a form of NH_4_NO_3_ and KNO_3_ to determine an amount of nitrogen application between two types at the same leaf biomass. Same size seedlings were planted under different treatments. Every treatment contained 30 uniform plants.

### 4.2. Treatments

Low nitrogen level: Flue-cured tobacco, (1) NL_G1, 4 mmol/L, HD; (2) NL_G2, 4 mmol/L, Z100; Burley tobacco, (3) NL_Y1, 4 mmol/L, TN90; (4) NL_Y2, 4 mmol/L, TN86.

High nitrogen level: Burley tobacco, (5) NH_Y1, 24 mmol/L, TN90; (6) NH_Y2, 24 mmol/L, TN86.

Composition of nutrient solution was as follows: N as NH_4_NO_3_, KNO_3_, Ca(NO_3_)_2_·4H_2_O, 1 mmol/L; P as NaH_2_PO_4_, 0.75 mmol/L; Ca as CaCl_2_·2H_2_O, 0.5 mmol/L; Mg as MgSO_4_·7H_2_O, 9 μmol/L; Mn as MnCl_2_·4H_2_O, 0.03 μmol/L; Mo as (NH_4_)_2_MoO_4_, 46 μmol/L; B as H_3_BO_3_, 8 μmol/L; Zn as ZnSO_4_·7H_2_O, 3 μmol/L; Cu as CuSO_4_·5H_2_O, 20 μmol/L; Fe as FeSO_4_·7H_2_O + Na_2_-EDTA. All nutrient solutions were continuously aerated with an air pumps. After germination, nutrient solution was replaced every six days. When seedlings were transplanted (sown 30 days later), nutrient solutions were refreshed every two days.

### 4.3. Sampling

Sampling and analysis were carried out 10 days after seedlings being transplanted (around 10:00 a.m.). Thirty uniform plants per treatment were divided into three groups. Fully expanded leaves (length > 5 cm, up to down, the fourth leaf from top) from the same position in three pots of each treatment were sampled in an ice box. Leave lamina between the middle sixth to eighth side vein were sliced into small sections for determination of NRA, GSA, NH_4_^+^ concentrations, pigment content and soluble protein content. The remaining 20 plants (per treatment) were used for the photosynthesis measurement by Li-6400 photosynthesis equipment (LI-COR Biotechnology, Lincoln, NE, USA) and Mini-PAM fluorometer (Walz, Effeltrich, Germany). Seedlings were then washed with flowing distilled water before being dried with absorbent paper and divided into root, stalk and leaves. Leaves of five plants from each treatment were mixed and frozen in liquid nitrogen immediately, and then kept at −80 °C. Tissues of 15 plants in one treatment were deactivated at 105 °C for 20 min and dried at 60 °C for 48 h. Dry matter of different tissues was weighed and grinded to pass through a screen with 60 meshes, and the final powder mixture was used to determine the nitrate, total nitrogen concentration, total soluble sugar content and soluble reducing sugar content in plant.

### 4.4. Measurement of NRA, GSA and Nitrate

Fresh lamina tissues without vein were cut into 2 mm × 5 mm pieces. NRA was measured based on the method described by Li [34]. GSA was determined according to the method described by O’Neal and Joy [35]. Nitrate content was determined according to the method described by Cataldo [36]. 

### 4.5. Measurement of Pigment Content, Photosynthetic Rate (Pn), Chlorophyll a Fluorescence

Pigment content was determined with 95% ethanol [37]. Photosynthetic rate (Pn) was observed using a portable photosynthesis system (LI-COR Biotechnology, 6400XT, Lincoln, NE, USA) at 9:00–11:00 a.m. as described by Liu [38]. Photosynthetic photon flux density (PPFD) and CO_2_ concentration in the reference chamber with a CO_2_ mixture were set as 1200 μmol m^−2^ s^−1^ and 400 μmol mol^−1^, respectively. Chlorophyll a fluorescence was measured in the same leaf determined nitrogen metabolism with a Mini-PAM fluorometer (Walz, Effeltrich, Germany) after a dark-adaptation of 20 min. Maximum quantum yield (Fv/Fm) was calculated according to the method described by Schreiber [39].

### 4.6. Measurement of Total Nitrogen, Total Soluble Sugar, Reducing Sugar Content

Total nitrogen, total soluble sugar and reducing sugar content were determined according to the method of modified Chinese Tobacco industry standard (YC/T 161, 159-2002). Samples (0.1 g powder mixture containing 0.1 g CuSO_4_ and 1 g K_2_SO_4_) were mixed with 5 mL concentrated sulphuric acid and retained 1–2 h at room temperature. The sample was kept for 150 °C (30 min), 250 °C (30 min), 370 °C (2 h) in furnace equipment (CIF, DS53-380, Los Angeles, CA, USA). After the mixture being cooled, 10 mL of deionized water was added to the sample and it was shaken thoroughly. The mixture being cooled 1–2 h was isochoric and filtered. The total nitrogen content in supernatant was determined with flow-injection-analysis (Bran + Luebbe, Hamburg, AA3, Germany). Samples (0.25 g powder mixture containing 25 mL 5% (*v*/*v*) acetic acid in 50 mL Erlenmeyer) were shaken for 30 min and filtered. The total soluble sugar and reducing sugar content in supernatant was determined with flow-injection-analysis (AA3).

### 4.7. RNA Extraction, Preparation of cDNA Library, and Sequencing

Total RNA was extracted using the mirVana miRNA Isolation Kit (Ambion, Waltham, MA, USA) following the manufacturer’s protocol. RNA integrity was evaluated using the Agilent 2100 Bioanalyzer (Agilent Technologies, Santa Clara, CA, USA). The samples with RNA Integrity Number (RIN) ≥7 were subjected to the subsequent analysis. The libraries were constructed using TruSeq Stranded mRNA LTSample Prep Kit (Illumina, San Diego, CA, USA) according to the manufacturer’s instructions. These libraries were then sequenced on the Illumina sequencing platform (HiSeqTM 2500 or Illumina HiSeq X Ten) and 125 bp/150 bp paired-end reads were generated. Quality control was assessed on the remaining reads using NGS QC Toolkit [40]. After removing low quality date, the clean reads with Q20 percentage of 94.07% were mapped to reference *P. trichocarpa* genome (ftp://ftp.solgenomics.net/genomes/Nicotiana_tabacum/assembly/Ntab-K326_AWOJ-SS.fa.gz) using bowtie2 or Tophat (http://tophat.cbcb.umd.edu/) [41,42].

### 4.8. RNA-Seq Analysis, GO and KEGG Pathway Enrichment Analysis of Differentially Expressed Genes (DEGs)

Transcript profiles of RNA-seq date were analyzed by calculating the read fragments per kilo base per million mapped reads (FPKM). FPKM value of each gene was calculated using cufflinks, and the read counts of each gene were obtained by htseq-count [43,44]. DEGs were identified using the DESeq (2012) functions estimate Size Factors and nbinomTest [45]. *p*-value < 0.05 and fold change > 2 or fold change < 0.5 was set as the threshold for significantly differential expression. Gene function was annotated based on databases of NR (NCBI non-redundant protein sequences), KOG (Clusters of Orthologous Groups of proteins) [46], Swiss-Prot (A manually annotated and reviewed protein sequence database) [47], KO (KEGG Ortholog database) [48], GO (Gene Ontology) [49]. Target genes of DEGs between flue-cured tobacco and burley tobacco in nitrogen and carbon metabolism were screened out by Short Time-series Expression Miner (STEM) version 1.3.8 (NIH, Bethesda, MD, USA) [50]. DEGs belonging to the same cluster were assumed to have similar expression pattern with each other [51]. GO enrichment and KEGG pathway enrichment analysis of DEGs were, respectively, achieved using R based on the hypergeometric distribution. Heatmaps analysis of DEGs was generated with R (3.4.1 version) (Lucent Technologies, Murray Hill, NJ, USA) pheatmap package [52]. Box plots were displayed according to the methods of Jin [53]. 

### 4.9. Validation by qRT-PCR Analysis

Quantification was performed with a two-step reaction process: reverse transcription (RT) and PCR. RT reactions were performed in a GeneAmp^®^ PCR System 9700 (Applied Biosystems, Foster, CA, USA) and GeneAmp^®^ PCR System 9700 (Applied Biosystems, Foster, CA, USA). Real-time PCR was performed using LightCycler^®^ 480 II Real-time PCR Instrument (Roche, Basel, Switzerland). Reactions were incubated in a 384-well optical plate (Roche, Basel, Switzerland) at 95 °C for 5 min, followed by 40 cycles of 95 °C for 10 s, 60 °C for 30 s. Each sample was run in triplicate for analysis. At the end of the PCR cycles, melting curve analysis was performed to validate the specific generation of the expected PCR product. The primer sequences were designed in the laboratory and synthesized by Generay Biotech (Shanghai, China) based on the mRNA sequences obtained from the NCBI database (Table S3). The expression levels of mRNAs were normalized to the expression in flue-cured tobacco (mean of HD and Z100) and were calculated using the 2^−ΔΔ*C*t^ method [54].

### 4.10. Statistical Analysis

Figures were processed using Origin Pro 9.0. (OriginLab Corporation, Northampton, MA, USA) and correlation analysis and variance between treatments were all processed using SPSS 20.0. (IBM, Palo Alto, CA, USA) Treatments were compared by LSD multiple range test (*p* < 0.05). All presented data is mean of three biological replicates (*n* = 3) and standard deviations were always less than 5% of data value.

## Figures and Tables

**Table 1:** Correlation coefficients between leaf matter accumulation and nitrogen and carbon metabolism in tobacco.

Parameter	NRA	GSA	NO_3_-N	TN	NO_3_-N/TN	Pigment	Pn
Total soluble sugar content	0.362	0.292	−0.452	−0.554	−0.486	0.433	0.777
Reducing sugar content	−0.119	−0.086	−0.805	−0.879 *	−0.825 *	0.019	0.368
Leaf biomass	0.631	0.796	0.226	0.014	0.201	0.822 *	0.738

NRA: Nitrate reductase activity; GSA: Glutamine synthetase activity; TN: Total nitrogen content; Pn: Photosynthetic rate. Symbol * indicates significant differences at 0.05, respectively.

**Table 2:** Correlation coefficients between nitrate content and enzymes of nitrogen assimilation, pigment content in tobacco.

Parameter	NRA/NA	GSA/NA	Pigment/NA	Pn/NA
NO_3_-N	−0.986 **	−0.828 *	−0.923 **	−0.970 **
NO_3_-N/TN	−0.989 **	−0.849 *	−0.936 **	−0.973 **

NA: Nitrogen application level; NRA/NA: Nitrate reductase activity/Nitrogen application level; GSA/NA: Glutamine synthetase activity/Nitrogen application level; Pn: Photosynthetic rate. Symbols ** and * indicate significant differences at 0.01 and 0.05, respectively.

## Supplementary Materials

Supplementary materials are available online, Table S1: Gene Table for Profile 38 (0.0, 0.0, −1.0, −1.0, −1.0, −1.0) correlated with carbon and nitrogen metabolism. Date are means of three biological replications. Table S2: Gene Table for Profile 73 (0.0, 0.0, 1.0, 1.0, 1.0, 1.0) correlated with carbon and nitrogen metabolism. Date are means of three biological replications; Table S3: The primers used in real-time PCR.

## References

[1] Henica F.S. (1932). The inheritance of the White Burley character in tobacco. Jpn. J. Crop Sci..

[2] Yang J., Tian Y.C., Yao X., Cao W.X., Zhang Y.S., Zhu Y. (2009). Hyperspectra estimation model for chlorophyll concentra tions in top leaves of rice. Acta Ecol. Sin..

[3] Shang Z.Q. (2007). Effects of nitrogen amount on growth and development yield and quality in burley tobacco. Chin. Agric. Sci. Bull..

[4] Sun W.S., Wang J., Zhou J., Ma Y.J., Yang H.J., Xu D.Y., Bai R.S., Jiao Z.H., Shi H.Z. (2015). Effect of nitrate nitrogen level in tobacco leaves on TSNAs formation during high temperature storage. Acta Tabacaria Sin..

[5] Reddy K.S., Menary R.C. (1990). Nitrate reductase and nitrate accumulation in relation to nitrate toxicity in Boronia megastigma. Physiol. Plant..

[6] Santamaria P. (2006). Nitrate in vegetables: Toxicity, content, intake and EC regulation. A review. J. Sci. Food Agric..

[7] Burton H., Dye N., Bush L. (1994). Relationship between tobacco-specific nitrosamines and nitrite from different air-cured tobacco varieties. J. Agric. Food Chem..

[8] Shi H.Z., Wang R.Y., Bush L.P. (2012). The relationships between TSNAs and their precursors in burley tobacco from different regions and varieties. J. Food Agric. Environ..

[9] Lewis R.S., Parker R.G., Danehower D.A., Andres K., Jack A.M., Whitley D.S., Bush L.P. (2012). Impact of Alleles at the Yellow Burley (Yb) Loci and Nitrogen Fertilization Rate on Nitrogen Utilization Efficiency and Tobacco-Specific Nitrosamine (TSNA) Formation in Air-Cured Tobacco. J. Agric. Food Chem..

[10] Vieira I.S., Vasconcelos E.P., Monteiro A.O.A. (1998). Nitrate accumulation, yield and leaf quality of turnip greens in response to nitrogen fertilization. Nutr. Cycl. Agroecosyst..

[11] Burns I.G., Zhang K., Turner M.K., Meacham M., Al-Redhiman K., Lynn J., Broadley M.R., Hand P., Pink D. (2011). Screening for genotype and environment effects on nitrate accumulation in 24 species of young lettuce. J. Sci. Food Agric..

[12] Tang Y.F., Sun X.C., Hu C.X. (2013). Genotypic differences in nitrate uptake, translocation and assimilation of two Chinese cabbage cultivars [*Brassica campestris* L. ssp. *Chinensis* (L.). Plant Physiol. Biochem..

[13] Man H.M., Boriel R., E1-Khatib R., Kirby E.G. (2005). Characterization of transgenic poplar with ectopic expression of pine cytosolic glutamine synthetase under conditions of varying nitrogen availability. New Phytol..

[14] Börner T., Mendel R.R., Schiemann J. (1986). Nitrate reductase is not accumulated in chloroplast-ribosome-deficient mutants of higher plants. Planta.

[15] Wallsgrove R.M., Lea P.J., Miflin B.J. (1979). Distribution of the enzymes of nitrogen assimilation within the pea leaf cell. Plant Physiol..

[16] Du J., Shu S., Shao Q.S. (2016). Mitigative effects of spermidine on photosynthesis and carbon nitrogen balance of cucumber seedlings under Ca(NO_3_)_2_ stress. J. Plant Res..

[17] Groben R., Kaloudas D., Raines C.A., Offmann B., Maberly S.C., Gontero B. (2010). Comparative sequence analysis of CP12, a small protein involved in the formation of a Calvin cycle complex in photosynthetic organisms. Photosynth. Res..

[18] Chen G., Yu R.B., Li N. (2004). EGY1 encodes a membrane-associated and ATP-independent metalloprotease that is required for chloroplast development. Plant J..

[19] Chen K.M., Holmstrom M., Raksajit W., Suorsa M., Piippo M., Aro E.M. (2010). Small chloroplasttargeted DnaJ proteins are involved in optimization of photosynthetic reactions in Arabidopsis thaliana. BMC Plant Biol..

[20] Jansson S. (1999). A guide to the Lhc genes and their relatives in Arabidopsis. Trends Plant Sci..

[21] Chourey P., Taliercio E., Carlson S., Ruan Y.L. (1998). Genetic evidence that the two isozymes of sucrose synthase present in developing maize endosperm are critical one for cell wall integrity and the other for starch biosynthesis. Mol. Gen. Genet..

[22] Chen S., Hajirezaei M., Börnke F. (2005). Differential expression of sucrose-phosphate synthase isoenzymes in tobacco reflects their functional specialization during dark-governed starch mobilization in source leaves. Plant Physiol..

[23] Volkert K., Debast S., Voll L.M., Voll H., Schie I., Hofmann J., Schneider S., Börnke F. (2014). Loss of the two major leaf isoforms of sucrose-phosphate synthase in Arabidopsis thaliana limits sucrose synthesis and nocturnal starch degradation but does not alter carbon partitioning during photosynthesis. J. Exp. Bot..

[24] Munoz F.J., Zorzano M.T.M., Alonso-Casajús N., Baroja-Fernandez E., Etxeberria E., Pozueta-Romero J. (2006). New enzymes, new pathways and an alternative view on starch biosynthesis in both photosynthetic and heterotrophic tissues of plants. Biocatal. Biotrans..

[25] Sun W.S., Yang J.J., Zhou J., Xu D., Bai R., Ma Y., Zhang G., Shi H. (2015). Effect of different nitrogen forms on nitrate nitrogen content and TSNAs formation in tobacco. Acta Tabacaria Sin..

[26] Zhong R.Q., Kays S., Schroeder B., Ye Z. (2002). Mutation of a chitinase-like gene causes ectopic deposition of lignin, aberrant cell shapes, and overproduction of ethylene. Plant Cell.

[27] Ai S.Y., Yao J.W., Huang X.H., Luo W., Ke Y., Ling D. (2002). Study on the nitrate reduction characteristic of vegetables. Plant Nutr. Fertil. Sci..

[28] Lin S.H., Kuo H.F., Canivenc G., Lin C.S., Lepetit M., Hsu P.K., Tillard P., Lin H.L., Wang Y.Y., Tsai C.B. (2008). Mutation of the Arabidopsis NRT1.5 nitrate transporter causes defective root-to-shoot nitrate transport. Plant Cell.

[29] Li W., Wang Y., Okamoto M., Crawford N.M., Siddiqi M.Y., Glass A.D. (2007). Dissection of the AtNRT2.1: AtNRT2.2 inducible high-affinity nitrate transporter gene cluster. Plant Physiol..

[30] Chen B.M., Wang Z.H., Li S.X. (2004). Effects of nitrate supply on plant growth, nitrate accumulation, metabolic nitrate concentration and nitrate reductase activity in three leafy vegetables. Plant Sci..

[31] Yanagisawa S. (2014). Transcription factors involved in controlling the expression of nitrate reductase genes in higher plants. Plant Sci..

[32] Yu L.H., Wu J., Tang H., Yuan Y., Wang S.M., Wang Y.P., Zhu Q.S., Li S.G., Xiang C.B. (2016). Overexpression of Arabidopsis NLP7 improves plant growth under both nitrogen-limiting and -sufficient conditions by enhancing nitrogen and carbon assimilation. Sci. Rep..

[33] Li Y.F., Chang D., Sun J., Yang H.J., Wang J., Shi H.Z. (2017). Difference of nitrogen metabolism between flue-cured tobacco and burley tobacco seedlings. Tob. Sci. Technol..

[34] Li H.S., Sun Q., Zhao S.J., Zhang W.H. (2000). Principle and Technology of Plant Physiological and Biochemical Experiments.

[35] O’Neal D., Joy K. (1974). Glutamine synthetase of pea leaves. I. Purification, stabilization, and pH optima. Plant Physiol..

[36] Cataldo D.A., Haroon M., Schrader L.E., Youngs V.L. (1975). Rapid cplorimetric Deternination of Nitrate in Plant-Tissure by Nitration of Salicylic Acid. Commun. Soil Sci. Plant Anal..

[37] Wintermans J.F.G.M., De Mots A. (1965). Spectrophotometric characteristics of chlorophylls a and b and their pheophytins in ethanol. Biochim. Biophys. Acta.

[38] Liu Y.F., Han X.R., Zhan X.M., Yang J.F., Wang Y.Z., Song Q.B., Chen X. (2013). Regulation of Calcium on Peanut Photosynthesis Under Low Night Temperature Stress. J. Integr. Agric..

[39] Schreiber U., Bilger W., Neubauer C., Schulze E.-D., Caldwell M.M. (1995). Chlorophyll Fluorescence as a Nonintrusive Indicator for Rapid Assessment of in Vivo Photosynthesis. Ecophysiology of Photosynthesis.

[40] Patel R.K., Jain M. (2012). NGS QC Toolkit: A Toolkit for Quality Control of Next Generation Sequencing Data. PLoS ONE.

[41] Langmead B., Salzberg S.L. (2012). Fast gapped-read alignment with Bowtie 2. Nat. Methods.

[42] Kim D., Pertea G., Trapnell C., Pimentel H., Kelley R., Salzberg S.L. (2013). TopHat2: Accurate alignment of transcriptomes in the presence of insertions, deletions and gene fusions. Genome Biol..

[43] Trapnell C., Roberts A., Goff L., Pertea G., Kim D., Kelley D.R., Pimentel H., Salzberg S.L., Rinn J.L., Pachter L. (2012). Differential gene and transcript expression analysis of RNA-seq experiments with TopHat and Cufflinks. Nat. Protoc..

[44] Anders S., Pyl P.T., Huber W. (2015). HTSeq-a Python framework to work with high-throughput sequencing data. Bioinformatics.

[45] Anders S., Huber W. (2013). Differential Expression of RNA-Seq Data at the Gene Level—The DESeq Package.

[46] Koonin E.V., Fedorova N.D., Jackson J.D., Jacobs A.R., Krylov D.M., Makarova K.S., Mazumder R., Mekhedov S.L., Nikolskaya A.N., Rao B.S. (2004). A comprehensive evolutionary classification of proteins encoded in complete eukaryotic genomes. Genome Biol..

[47] Apweiler R., Bairoch A., Wu C.H., Barker W.C., Boeckmann B., Ferro S., Gasteiger E., Huang H.Z., Lopez R., Magrane M. (2004). UniProt: The Universal Protein knowledgebase. Nucleic Acids Res..

[48] Kanehisa M., Goto S., Kawashima S., Okuno Y., Hattori M. (2004). The KEGG resource for deciphering the genome. Nucleic Acids Res..

[49] Ashburner M., Ball C.A., Blake J.A., David B., Butler H., Cherry J.M., Davis A.P., Dolinski K., Dwight S.S., Eppig J.T. (2000). Gene Ontology: Tool for the unification of biology. Nat. Genet..

[50] Ernst J., Bar-Joseph Z. (2006). STEM: A tool for the analysis of short time series gene expression data. BMC Bioinform..

[51] Zhou Q., Guo J.J., He C.T., Shen C., Huang Y.Y., Chen J.X., Guo J.H., Yuan J.G., Yang Z.Y. (2016). Comparative Transcriptome Analysis between Low- and High-Cadmium-Accumulating Genotypes of Pakchoi (*Brassica chinensis* L.) in Response to Cadmium Stress. Environ. Sci. Technol..

[52] Britton N.F., Lin X.H., Safer H.M., Schneider M.V., Singh M., Tramontano A. (2014). RNA-Seq Data Analysis-A Practical Approach.

[53] Jin J.J., Sun Y.W., Qu J., Syah R., Lim C., Alfiko Y., Rahman N., Suwanto A., Yue G.H., Wong L. (2017). Transcriptome and functional analysis reveals hybrid vigor for oil biosynthesis in oil palm. Sci. Rep..

[54] Livak K.J., Schmittgen T.D. (2001). Analysis of relative gene expression data using real-time quantitative PCR and the 22DDCT method. Methods.

